# Prognostic value of tumor deposits and positive lymph node ratio in stage III colorectal cancer: a retrospective cohort study

**DOI:** 10.1097/JS9.0000000000001295

**Published:** 2024-03-18

**Authors:** Lei Liu, Jie Ji, Xianxiu Ge, Zuhong Ji, Jiacong Li, Jie Wu, Juntao Zhu, Jianan Yao, Fangyu Zhu, Boneng Mao, Zhihong Cao, Jinyi Zhou, Lin Miao, Guozhong Ji, Dong Hang

**Affiliations:** a Medical Centre for Digestive Diseases, The Second Affiliated Hospital of Nanjing Medical University; b Department of Non-Communicable Chronic Disease Control, Jiangsu Provincial Center for Disease Control and Prevention; c Jiangsu Key Lab of Cancer Biomarkers, Prevention and Treatment, Collaborative Innovation Center for Cancer Personalized Medicine and China International Cooperation Center for Environment and Human Health, Gusu School; d Department of Epidemiology, School of Public Health, Nanjing Medical University, Nanjing; e Department of Gastroenterology, The Affiliated Yixing Hospital of Jiangsu University, Yixing; f Department of General Surgery, The First Affiliated Hospital of Nanjing Medical University, Nanjing, Jiangsu Province, People’s Republic of China

**Keywords:** colorectal cancer, laterality, lymph node ratio, prognosis, stage III, tumor deposits

## Abstract

**Background:**

In colorectal cancer (CRC), tumor deposits (TD) have been used to guide the N staging only in node-negative patients. It remains unknown about the prognostic value of TD in combination with positive lymph node ratio (LNR) in stage III CRC.

**Patients and methods:**

The authors analyzed data from 31 139 eligible patients diagnosed with stage III CRC, including 30 230 from the Surveillance, Epidemiology, and End Results (SEER) database as a training set and 909 from two Chinese hospitals as a validation set. The associations of TD and LNR with cancer-specific survival (CSS) and overall survival (OS) were evaluated using the Kaplan–Meier method and Cox regression models.

**Results:**

Both TD-positive and high LNR (value ≥0.4) were associated with worse CSS in the training [multivariable hazard ratio (HR), 1.50; 95% CI: 1.43–1.58 and HR, 1.74; 95% CI: 1.62–1.86, respectively] and validation sets (HR, 1.90; 95% CI: 1.41–2.54 and HR, 2.01; 95% CI: 1.29–3.15, respectively). Compared to patients with TD-negative and low LNR (value<0.4), those with TD-positive and high LNR had a 4.09-fold risk of CRC-specific death in the training set (HR, 4.09; 95% CI: 3.54–4.72) and 4.60-fold risk in the validation set (HR, 4.60; 95% CI: 2.88–7.35). Patients with TD-positive/H-LNR CRC on the right side had the worst prognosis (*P*<0.001). The combined variable of TD and LNR contributed the most to CSS prediction in the training (24.26%) and validation (32.31%) sets. A nomogram including TD and LNR showed satisfactory discriminative ability, and calibration curves indicated favorable consistency in both the training and validation sets.

**Conclusions:**

TD and LNR represent independent prognostic predictors for stage III CRC. A combination of TD and LNR could be used to identify those at high-risk of CRC deaths.

## Introduction

Highlights

- Tumor deposits (TD) have been utilized to guide N staging without considering metastatic lymph nodes and the total number of retrieved lymph nodes.
- In this study, we analyzed the prognostic value of TD in combination with positive lymph node ratio (LNR) in stage III CRC.
- Both TD and LNR are independent prognostic factors in stage III colorectal cancer.
- The combination of TD and LNR holds potential to improve the prognosis prediction, especially for right-sided CRC.

Colorectal cancer (CRC) is the second leading cause of cancer-related deaths worldwide^1^, with a 5-year overall survival rate ranging from 92% for stage I to a dismal 11% for stage IV^2,3^. Although the TNM stage is currently used to guide treatment, its accuracy is limited by the heterogeneity of stage III CRC in terms of tumor invasion depth and the number of lymph node metastases (LNM). The International Duration Evaluation of Adjuvant Chemotherapy (IDEA) has categorized stage III colon cancer into high-risk group (T4 or/and N2) and low-risk group (T1-3 N1) based on chemotherapy duration^2,4^. However, ~30% of stage III CRC cases will experience recurrence, with the majority eventually succumbing to the disease due to disease progression^5^. Hence, it is imperative to identify new predictive markers for stage III CRC.

In the 8th edition UICC TNM classification, tumor deposits (TD) referred to discrete nodules of cancer in the lymph drainage area of pericolorectal adipose tissue, without histological evidence of neural structures, vessels, and lymph. The presence of TD without metastatic lymph nodes is classified as N1c and has been considered as an independent prognostic indicator in CRC^6^. Integrating the number of TD and LNM for restaging N can help assess prognosis and guide treatment^7,8^. However, the strategy had drawbacks in that it simply treated TD as a lymph node^9^. In addition, the prognostic value of TD appeared to be more significant for patients with N0/N1 stage but not for those with N2 stage^10^. Furthermore, these studies did not take into account the number of resected lymph nodes, which may also affect the prognosis. It is possible that the positive lymph node ratio (LNR), which was defined as the ratio of LNM to the total number of retrieved lymph nodes, could exhibit superior performance in predicting prognosis compared to LNM alone^11–13^.

Moreover, in metastatic CRC, right-sided tumors had been linked to female sex, older age, mucinous histology, KRAS mutations, and worse outcomes compared to left-sided tumors^14^. However, studies in nonmetastatic CRC seemed to show no association between tumor laterality and survival^15^. There was contradictory evidence regarding the association between tumor laterality and patients’ survival in CRC.

To date, no reports have evaluated the prognostic value of TD considering both metastatic and total retrieved lymph nodes. Therefore, we conducted a retrospective cohort study to assess the prognostic value of TD and LNR, and performed stratified analyses according to tumor laterality in stage III CRC.

## Material and methods

### Patients and study design

This was a retrospective cohort study with two datasets. The training set comprised data from the SEER database between 2010 and 2019 (http://seer.cancer.gov/seerstat/). The validation set was collected continuously from two Chinese hospitals, including 372 patients from a tertiary hospital in Nanjing between January 2015 and December 2020, and 537 patients from a tertiary hospital in Yixing between January 2013 and December 2020. Patients included in the study met the following criteria: (1) Stage III CRC patients; (2) Those who underwent surgical resection and were confirmed microscopically; (3) Primary site ICD-O-3 codes: C18.0, C18.1, C18.2, C18.3, C18.4, C18.5, C18.6, C18.7, C18.8, C18.9, C19.9, and C20.9; and (4) Histological ICD-O-3 codes: 8140-8147, 8210-8211, 8220-8221, 8260-8263, 8480-8481, and 8490^16^. Exclusion criteria included: (1) Nonprimary tumors; (2) Tumors located in the appendix; (3) Patients younger than 18 years old; (4) Those with survival times of less than 1 month; and (5) Individuals with incomplete or incorrect clinicopathological information. The flow chart is shown in Fig. S1 (Supplemental Digital Content 2, http://links.lww.com/JS9/C110).

The study was approved by the Ethics Committee of the two Chinese hospitals. The Strengthening the Reporting of Cohort Studies in Surgery (STROCSS) criteria^17^ (Supplemental Digital Content 1, http://links.lww.com/JS9/C109) was followed. Informed consent was waived by the two committees due to the retrospective nature of the study.

### Study variables

The clinicopathological characteristics were collected from medical records, including age, sex, registry, year of diagnosis, tumor laterality, tumor size, tumor number, T stage, N stage, metastatic lymph nodes, total examined lymph nodes (examined N), histology, grade, TD, carcinoembryonic antigen (CEA), perineural invasion (PNI), neoadjuvant therapy, and chemotherapy. TD and LNR were obtained from the final histopathology report. Pathology slides of patients from two Chinese hospitals were cross-reviewed by two senior pathologists. All clinicopathological factors were assessed according to the 8th edition UICC classification^6^. Right-sided tumors were defined as lesions from cecum to transverse colon, while left-sided tumors were defined as lesions from splenic flexure to rectum^18^.

Survival outcomes in the training set were acquired from the SEER database, while survival outcomes in the validation set were obtained from both telephone-based active follow-up and China’s mortality registration system. CSS was measured from the date of diagnosis until death due to CRC, and OS was measured as the interval from diagnosis to death of any cause.

### Statistical analysis

The *χ*^2^ test was used for categorical variables, and the Wilcoxon rank-sum test was used for continuous variables. We utilized X-tile software (version 3.5.0; Yale University) to determine the optimal cutoff value for LNR in the training set. The value was identified by scoring the maximum X-squared value in the Kaplan–Meier test based on survival time and outcomes^19,20^. Subsequently, patients were categorized into low LNR (L-LNR) and high LNR (H-LNR) groups based on the determined cutoff. Survival analyses were conducted using the Kaplan–Meier method and the log-rank test. We calculated the *P*-value of heterogeneity using the contrast test method to assess the impact of the study variable on prognosis stratified by tumor laterality. Significant univariable variables were included in the multivariable Cox regression model. A sensitivity analysis was performed by additionally adjusting for histology (adenocarcinoma, mucinous adenocarcinoma, signet ring cell carcinoma), diagnosis year (continuous), and radiation (no, yes). Moreover, we performed a sensitivity analysis by excluding those who received neoadjuvant treatment. We also performed a stratified analysis according to the year of CRC diagnosis. The relative contribution of individual or combined variable to CSS was assessed by multivariable Cox regression models.

We constructed a nomogram and evaluated its discriminative ability using the time-dependent receiver operating characteristic (ROC) curve and the area under the curve (AUC). Additionally, the calibration of the model was evaluated through calibration curves and the Hosmer–Lemeshow goodness-of-fit test^21^. A two-tailed *P*-value less than 0.05 indicated statistical significance. All statistical analyses were performed using SPSS version 25.0, GraphPad Prism 9.4.1, and R version 3.6.1.

## Results

### Patient baseline characteristics

A total of 30 230 patients were eligible for the training set, and 909 patients were included in the validation set. The median ages at CRC diagnosis in the two sets were 64 (IQR, 54–74) and 66 (IQR, 58–74) years, respectively. The corresponding median follow-up times were 38 (IQR, 17–67) and 38 (IQR, 22–61) months. The 5-year CSS rates were 72.6 and 70.8%, while 5-year OS rates were 64.9 and 63.4%, respectively. Baseline characteristics of patients according to TD and LNR are shown in Table 1. In the training set, 7909 patients (26.2%) had TD, among whom 2996 (37.9%) had pN1a/b, 1,774 (22.4%) had pN1c, and 3,139 (39.7%) had pN2 stage tumors. In addition, 3794 patients (12.6%) had H-LNR, of whom 233 (6.1%) had pN1a/b and 3561 (93.9%) had pN2. In the validation set, TD-positive was detected in 247 patients (27.2%), while H-LNR were identified in 147 (16.2%) patients. Patients with TD-positive and H-LNR were more likely to have poor differentiation, T3-4 stage, and the presence of PNI compared with patients lacking these features in the two sets (*P*<0.01).

**Table 1:** Patients’ characteristics by tumor deposits and lymph node ratio in two cohorts.

	Training set			Validation set			Training set			Validation set		
Characteristic^a^ (*N*, %)	TD-negative (*N*=22 321)	TD-positive (*N*=7909)	*P* ^b^	TD-negative (*N*=662)	TD-positive (*N*=247)	*P* ^b^	Low LNR (*N*=26 436)	High LNR (*N*=3794)	*P* ^b^	Low LNR (*N*=762)	High LNR (*N*=147)	*P* ^b^
Age			0.89			0.51			0.25			0.003
Mean (SD)	63.89 (13.81)	63.52 (13.96)		65.2 (11.97)	65.36 (11.53)		63.77 (13.77)	63.94 (14.39)		65.79 (11.41)	62.43 (13.54)	
Q1–Q3	54.0–74.0	54.0–74.0		58.0–74.0	58.0–74.0		54.0–74.0	54.0–75.0		59.0–74.0	54.0–72.0	
Age			0.40			0.29			0.07			0.12
≤65 years	12 055 (54.0)	4315 (54.6)		317 (47.4)	127 (51.4)		14 368 (54.4)	2002 (52.8)		361 (47.4)	80 (54.4)	
>65 years	10 266 (46.0)	3594 (45.4)		348 (52.6)	120 (48.6)		12 068 (45.6)	1792 (47.2)		401 (52.6)	67 (45.6)	
Sex			0.01			0.92			0.06			0.35
Female	10 829 (48.5)	3705 (46.8)		284 (42.9)	105 (42.5)		12 764 (48.3)	1770 (46.7)		321 (42.1)	68 (46.3)	
Male	11 492 (51.5)	4204 (53.2)		378 (57.1)	142 (57.5)		13 672 (51.7)	2024 (53.3)		441 (57.9)	79 (53.7)	
Laterality			<0.001			0.10			0.50			0.01
Left	11 722 (52.5)	4704 (59.5)		465 (70.2)	187 (75.7)		14 345 (54.3)	2081 (54.8)		534 (70.1)	118 (80.3)	
Right	10 599 (47.5)	3205 (40.5)		197 (29.8)	60 (24.3)		12 091 (45.7)	1713 (45.2)		228 (29.9)	29 (19.7)	
Tumor number			0.92			0.64			0.68			0.70
1	21 980 (98.5)	7787 (98.5)		637 (96.2)	236 (95.5)		26 034 (98.5)	3733 (98.4)		731 (95.9)	142 (96.6)	
>1	341 (1.5)	122 (1.5)		25 (3.8)	11 (4.5)		402 (1.5)	61 (1.6)		31 (4.1)	5 (3.4)	
Tumor size			<0.001			0.45			<0.001			0.13
≤5 cm	14 063 (63.0)	4706 (59.5)		518 (78.2)	199 (80.6)		16 578 (62.7)	2191 (57.7)		608 (79.8)	109 (74.1)	
>5 cm	8258 (37.0)	3203 (40.5)		144 (21.8)	48 (19.4)		9858 (37.3)	1603 (42.3)		154 (20.2)	38 (25.9)	
Grade			<0.001			0.01			<0.001			<0.001
Well/moderate	17 488 (78.3)	5781 (73.1)		485 (73.3)	159 (64.4)		21 040 (79.6)	2229 (58.8)		575 (75.5)	69 (46.9)	
Poor/undifferentiated	4833 (21.7)	2128 (26.9)		177 (26.7)	88 (35.6)		5396 (20.4)	1565 (41.2)		187 (24.5)	78 (53.1)	
T stage			<0.001			0.03			<0.001			0.04
T1-2	3442 (15.4)	434 (5.5)		81 (12.2)	18 (7.3)		3664 (13.9)	212 (5.6)		90 (11.8)	9 (6.1)	
T3-4	18 879 (84.6)	7475 (94.5)		581 (87.8)	229 (92.7)		22 772 (86.1)	3582 (94.4)		672 (88.2)	138 (93.9)	
N stage			<0.001			0.48			<0.001			<0.001
N1 (<4 nodes)	15 593 (69.9)	4770 (60.3)		420 (63.4)	163 (66.0)		20 130 (76.1)	233 (6.1)		582 (76.4)	1 (0.7)	
N2 (≥4 nodes)	6728 (30.1)	3139 (39.7)		242 (36.6)	84 (34.0)		6306 (23.9)	3561 (93.9)		180 (23.6)	146 (99.3)	
Examined N			<0.001			0.67			<0.001			0.14
<12	1726 (7.7)	842 (10.6)		66 (10.0)	27 (10.9)		1819 (6.9)	749 (19.7)		73 (9.6)	20 (13.6)	
≥12	20 595 (92.3)	7067 (89.4)		596 (90.0)	220 (89.1)		24 617 (93.1)	3045 (80.3)		689 (90.4)	127 (86.4)	
CEA			<0.001			0.20			<0.001			0.66
Negative	13 225 (59.2)	4097 (51.8)		393 (59.4)	135 (54.7)		15 410 (58.3)	1912 (50.4)		445 (58.4)	83 (56.5)	
Positive	9096 (40.8)	3812 (48.2)		269 (40.6)	112 (45.3)		11 026 (41.7)	1882 (49.6)		317 (41.6)	64 (43.5)	
PNI			<0.001			<0.001			<0.001			<0.001
Absent	18 873 (84.6)	5369 (67.9)		532 (80.4)	166 (67.2)		21 666 (82.0)	2576 (67.9)		633 (83.1)	65 (44.2)	
Present	3448 (15.4)	2540 (32.1)		130 (19.6)	81 (32.8)		4770 (18.0)	1218 (32.1)		129 (16.9)	82 (55.8)	
Neoadjuvant therapy			0.000			0.18			0.03			0.77
No	20 653 (92.5)	7128 (90.1)		629 (95.0)	229 (92.7)		24 329 (92.0)	3452 (91.0)		720 (94.5)	138 (93.9)	
Yes	1668 (7.5)	781 (9.9)		33 (5.0)	18 (7.3)		2107 (8.0)	342 (9.0)		42 (5.5)	9 (6.1)	
Chemotherapy			0.04			0.99			0.75			0.15
No	5664 (25.4)	2098 (26.5)		265 (40.0)	99 (40.1)		6796 (25.7)	966 (25.5)		313 (41.1)	51 (34.7)	
Yes	16 657 (74.6)	5811 (73.5)		397 (60.0)	148 (59.9)		19 640 (74.3)	2828 (74.5)		449 (58.9)	96 (65.3)	
LNR			<0.001			0.22			NA			NA
Low	20 007 (89.6)	6429 (81.3)		561 (84.7)	201 (81.4)		NA	NA		NA	NA	
High	2314 (10.4)	1480 (18.7)		101 (15.3)	46 (18.6)		NA	NA		NA	NA	
TD			NA			NA			<0.001			0.22
Negative	NA	NA		NA	NA		20 007 (75.7)	2314 (61.0)		561 (73.6)	101 (68.7)	
Positive	NA	NA		NA	NA		6429 (24.3)	1480 (39.0)		201 (26.4)	46 (31.3)	

a Values are presented in mean (SD) or IQR for continuous variables and counts (percentages) for the categorical variables.

b Categorical variables were assessed using the *χ*^2^ test, while continuous variables by the Wilcoxon rank-sum test.

CEA, carcinoembryonic antigen; Examined N, total examined lymph nodes; LNR, lymph node ratio; NA, not applicable; PNI, perineural invasion; Q1–Q3, first quartile-third quartile; TD, tumor deposit.

### Association of TD and lymph node ratio with prognosis

**Figure 1:**
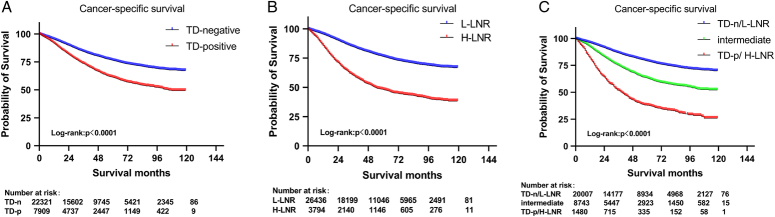
Kaplan–Meier plots of CSS by TD (A), LNR (B), and the combined variable of TD and LNR (C) in the training set. CSS, cancer-specific survival; LNR, lymph node ratio; L-LNR, low LNR; H-LNR, high LNR; TD, tumor deposit; TD-n, TD-negative; TD-p, TD-positive.

TD-positive and H-LNR were both associated with worse CSS (Fig. 1A, B) and OS (Fig. S2A and B, Supplemental Digital Content 2, http://links.lww.com/JS9/C110) in the training (all *P-*value <0.001) and validation sets (all *P-*value<0.001; Fig. S3A-D, Supplemental Digital Content 2, http://links.lww.com/JS9/C110). Furthermore, the combined variable of TD and LNR was classified into three subgroups: TD-negative/L-LNR, intermediate (TD-positive/L-LNR or TD-negative/H-LNR) and TD-positive/H-LNR. The results showed that the combined variable effectively stratified the prognosis, with 5-year CSS of 79.0, 63.2, and 37.9% in the training set and 78.9, 60.2, 44.5% in the validation set, respectively (*P*<0.001; Fig. 1C and Fig. S3E, Supplemental Digital Content 2, http://links.lww.com/JS9/C110). Compared to patients with TD-negative/L-LNR, those with TD-positive/H-LNR had a 4.09-fold risk of CRC-specific death in the training set (HR, 4.09; 95% CI: 3.54–4.72; *P*<0.001) and 4.60-fold risk in the validation set (HR, 4.60; 95% CI: 2.88–7.35; *P*<0.001). The results were consistent for OS (*P*<0.01; Fig. S2C, Supplemental Digital Content 2, http://links.lww.com/JS9/C110 and Fig. S3F, Supplemental Digital Content 2, http://links.lww.com/JS9/C110). Regarding the intermediate subgroup, patients with the TD-negative/H-LNR exhibited a worse prognosis compared to those with TD-positive/L-LNR in the training set (HR, 1.52; 95% CI: 1.31–1.76; *P*<0.05; Figure S4A, Supplemental Digital Content 2, http://links.lww.com/JS9/C110). Similar pattern was observed in the validation set (Figure S4B, Supplemental Digital Content 2, http://links.lww.com/JS9/C110). Importantly, when patients were stratified into low- (T1-3 N1) and high-risk (T4 and/or N2) groups in the training set, the 5-year CSS rates were 84.1, 74.7, and 49.7% among low-risk (T1-3N1) group (*P*<0.001; Fig. S5A, Supplemental Digital Content 2, http://links.lww.com/JS9/C110), while 69.5, 55.0, and 37.2% among high-risk (T4 and/or N2) group (*P*<0.001; Fig. S5C, Supplemental Digital Content 2, http://links.lww.com/JS9/C110), respectively. In both risk groups, patients with TD-positive/H-LNR tumors had the worst CSS. Notably, the 5-year CSS of low-risk tumors patients with TD-positive/H-LNR was worse than those of high-risk tumors with TD-negative/L-LNR (49.7 versus 69.5%; *P*<0.001). The results show similarity in terms of OS (*P*<0.001; Fig. S5B and D, Supplemental Digital Content 2, http://links.lww.com/JS9/C110).

### Prognostic value of TD and lymph node ratio according to tumor laterality

Kaplan–Meier analysis revealed that in the training set, both TD-positive and H-LNR patients had worse CSS in the left-sided and right-sided subgroups (Fig. S6A-D, Supplemental Digital Content 2, http://links.lww.com/JS9/C110). The assessment of *P*-value heterogeneity indicated a stronger association between LNR and right-sided colon cancer (HR, 3.09; 95% CI: 2.86–3.34; *P*<0.001) compared to left-sided tumors (HR, 2.47; 95% CI: 2.28–2.68; *P*<0.001) (*P*-value for heterogeneity <0.001). However, the difference in the association between LNR and CSS was not observed in the validation set (left-sided tumors: HR, 3.13; 95% CI: 2.20–4.45; *P*<0.001; right-sided tumors: HR, 1.70; 95% CI, 0.87–3.34; *P*=0.12; *P*-value for heterogeneity=0.12), likely due to the relatively small sample size in each subgroup. Furthermore, although TD had significant prognostic significance in both left-sided and right-sided tumors, there was no difference between the two subgroups (left-sided tumors: HR, 1.80; 95% CI: 1.68–1.93; *P*<0.001; right-sided tumors: HR, 1.93; 95% CI: 1.80–2.08; *P*<0.001; *P-*value for heterogeneity=0.18). The results were consistent for OS (Supplementary Table 1, Supplemental Digital Content 2, http://links.lww.com/JS9/C110).

**Figure 2:**
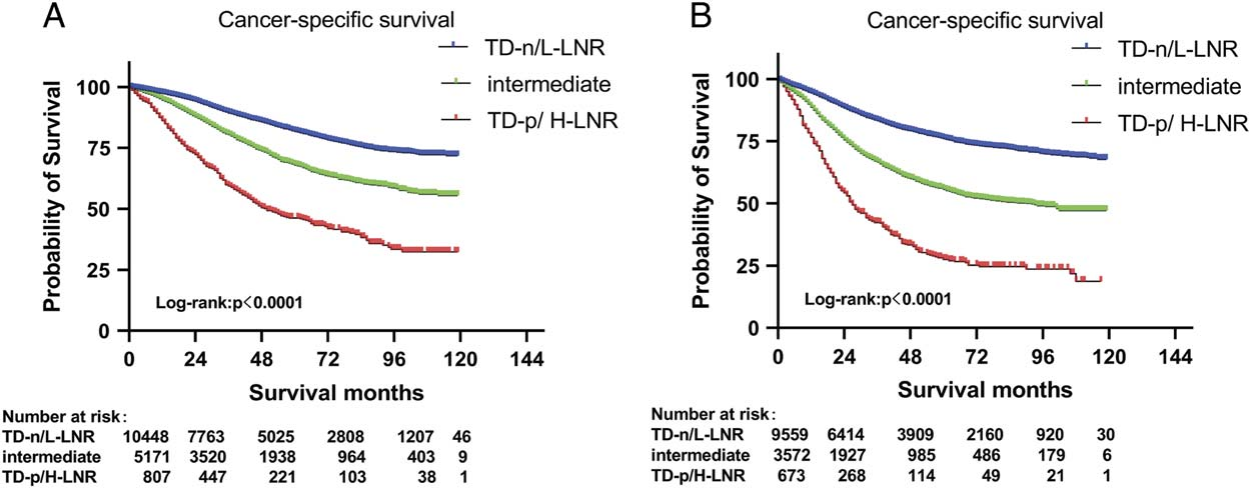
CSS for the combined variable in left-sided (A) and right-sided subgroups (B). CSS, cancer-specific survival; LNR, lymph node ratio; L-LNR, low LNR; H-LNR, high LNR; TD, tumor deposit; TD-n, TD-negative; TD-p, TD-positive.

In the training set, when stratified by tumor laterality, the combined variable (TD-negative/L-LNR, intermediate, and TD-positive/H-LNR) still significantly stratified the prognosis. The 5-year CSS rates in the left-sided subgroup were 81.7, 68.0, and 46.3%, respectively (*P*<0.001; Fig. 2A), and in the right-sided subgroup were 76.0, 55.7, and 27.4%, respectively (*P*<0.001; Fig. 2B). Notably, patients with TD-positive/H-LNR on the right side had the worst prognosis, whereas those with TD-negative/L-LNR on the left side had a relatively better prognosis, with 5-year CSS rates of 27.4 versus 81.7% (HR, 7.04; 95% CI: 5.75–8.61; *P*<0.001; Fig. S6E, Supplemental Digital Content 2, http://links.lww.com/JS9/C110).

### Relative contribution of individual or combined variable in predicting CSS and the establishment of a nomogram prediction model

**Figure 3:**
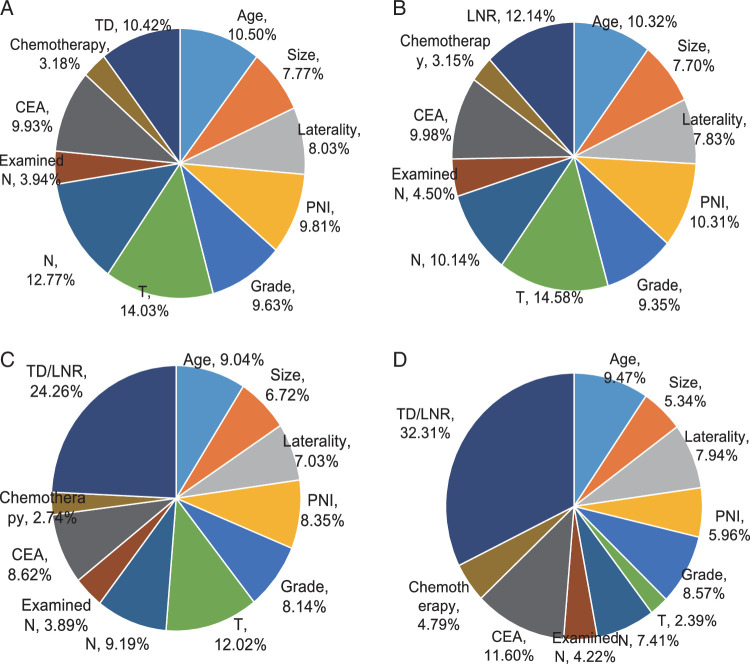
Relative contribution of TD (A), LNR (B), and the combined variable to CSS in the training (C) and validation set (D). CSS, cancer-specific survival; CEA, carcinoembryonic antigen; Examined N, total examined lymph nodes; LNR, lymph node ratio; PNI, perineural invasion; TD, tumor deposit.

TD and LNR were both positively associated with worse CSS in multivariable Cox regression models after adjusting for age, tumor size, laterality, grade, T stage, N stage, examined N, CEA, PNI, and chemotherapy in the two sets (*P*<0.01; Table 2, Supplementary Table 2, Supplemental Digital Content 2, http://links.lww.com/JS9/C110). The results were consistent for OS (Supplementary Table 2, Supplemental Digital Content 2, http://links.lww.com/JS9/C110 and 3, Supplemental Digital Content 2, http://links.lww.com/JS9/C110). The results were stable when additionally adjusting for histology, diagnosis year, and radiation (Supplementary table 4, Supplemental Digital Content 2, http://links.lww.com/JS9/C110). The sensitivity analysis by excluding those who received neoadjuvant treatment showed that the associations of TD and LNR with CSS and OS were basically unchanged (Supplementary Table 5, Supplemental Digital Content 2, http://links.lww.com/JS9/C110 and 6, Supplemental Digital Content 2, http://links.lww.com/JS9/C110). We also found that the prognostic associations for TD and LNR were consistent across the year of CRC diagnosis in the training and validation sets (Supplementary Table 7, Supplemental Digital Content 2, http://links.lww.com/JS9/C110) (*P* for heterogeneity>0.05). We further analyzed the relative contributions of individual or combined variable to CSS using multivariable models. In the model of the training set with TD, the top four contributors to CSS were T stage (14.03%), N stage (12.77%), age (10.50%), and TD (10.42%) (Fig. 3A). In the model with LNR, LNR ranked second (12.14%) behind T stage (14.58%), and surpassed age (10.32%) and PNI (10.31%) (Fig. 3B). Moreover, in the model with the combined variable of TD and LNR, it contributed the most (24.26%) to CSS, followed by T stage (12.02%), N stage (9.19%), and age (9.04%) (Fig. 3C). Similarly, the combined variable made the largest contribution in the validation set, accounting for 32.31% (Fig. 3D).

**Table 2:** Univariable and multivariable analyses of cancer-specific survival in the training set.

	Univariable^a^		Multivariable^b^	
Variables	HR (95% CI)	*P*	HR (95% CI)	*P*
Age	1.03 (1.03–1.03)	<0.001	1.02 (1.02–1.02)	<0.001
Sex				
Female	1 (Reference)			
Male	1.02 (0.97–1.07)	0.44		
Laterality				
Left	1 (Reference)		1 (Reference)	
Right	1.40 (1.33–1.47)	<0.001	1.14 (1.08–1.20)	<0.001
Tumor number				
1	1 (Reference)			
>1	1.21 (1.00–1.45)	0.05		
Tumor size				
≤5 cm	1 (Reference)		1 (Reference)	
>5 cm	1.39 (1.33–1.46)	<0.001	1.15 (1.10–1.21))	<0.001
Grade				
Well/moderate	1 (Reference)		1 (Reference)	
Poor/undifferentiated	1.82 (1.73–1.92)	<0.001	1.37 (1.29–1.44)	<0.001
T stage				
T1-2	1 (Reference)		1 (Reference)	
T3-4	3.02 (2.72–3.36)	<0.001	2.03 (1.82–2.27)	<0.001
N stage				
N1 (<4 nodes)	1 (Reference)		1 (Reference)	
N2 (≥4 nodes)	2.03 (1.93–2.13)	<0.001	1.53 (1.44–1.62)	<0.001
Examined N				
<12	1 (Reference)		1 (Reference)	
≥12	0.67 (0.63–0.72)	<0.001	0.69 (0.64–0.74)	<0.001
CEA				
Negative	1 (Reference)		1 (Reference)	
Positive	1.75 (1.66–1.83)	<0.001	1.45 (1.38–1.52)	<0.001
PNI				
Absent	1 (Reference)		1 (Reference)	
Present	1.78 (1.69–1.88)	<0.001	1.44 (1.36–1.52)	<0.001
Neoadjuvant therapy				
No	1 (Reference)			
Yes	1.07 (0.99–1.16)	0.08		
Chemotherapy				
No	1 (Reference)		1 (Reference)	
Yes	0.43 (0.41–0.45)	<0.001	0.51 (0.48–0.54)	<0.001
TD				
Negative	1 (Reference)		1 (Reference)	
Positive	1.80 (1.71–1.90)	<0.001	1.50 (1.43–1.58)	<0.001
LNR				
Low	1 (Reference)		1 (Reference)	
High	2.74 (2.59–2.90)	<0.001	1.74 (1.62–1.86)	<0.001

a Univariable Cox proportional hazards regression models.

b Multivariable Cox proportional hazards regression model included age, laterality, tumor size, grade, T stage, N stage, examined N, CEA, PNI, chemotherapy, TD, and LNR.

CEA, carcinoembryonic antigen; Examined N, total examined lymph nodes; HR, hazard ratio; LNR, lymph node ratio; PNI, perineural invasion; TD, tumor deposit.

**Figure 4:**
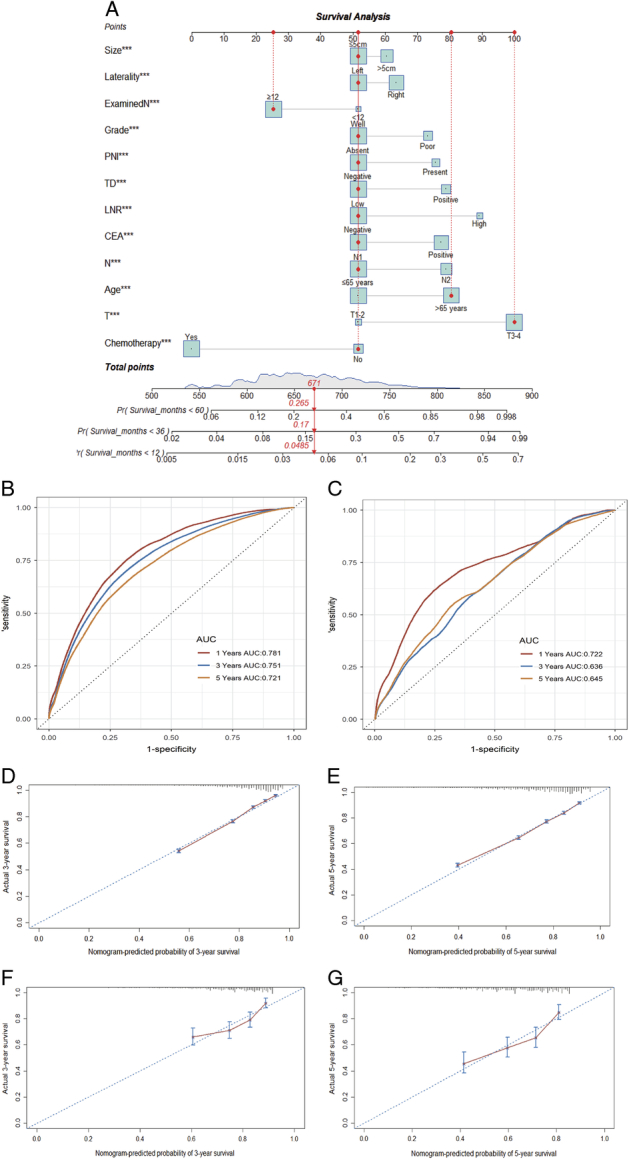
The nomogram model (A) and the ROC curves with AUC values for the training (B) and validation sets (C). The calibration curves showed the predicted and observed CSS probabilities in the training (D and E) and validation (F and G) sets. AUC, the area under the curve; CSS, cancer-specific survival. CEA, carcinoembryonic antigen; Examined N, total examined lymph nodes; LNR, lymph node ratio; PNI, perineural invasion; ROC, the receiver operating characteristic curve; TD, tumor deposit.

A nomogram was constructed based on significant variables from the multivariable Cox model (Fig. 4A), demonstrating acceptable discriminative capabilities with AUC values of 0.75 and 0.72 for 3-year and 5-year CSS in the training set, and 0.64 and 0.65 in the validation set, respectively (Fig. 4B and C). Furthermore, calibration curves exhibited favorable consistency between the predicted and observed CSS probabilities in the training set (Figs. 4D and E), with the X-squared value of 10.35 and the corresponding *P*-value of 0.24 (>0.05). The similar results were observed in the validation set (Figs. 4F and G), with the X-squared value of 15.12 and the corresponding *P*-value of 0.06 (>0.05).

## Discussion

N1c represented the presence of TD without metastatic lymph nodes and had similar prognostic efficacy to N1a/b. Integrating the number of TD and LNM to regain N staging, patients initially classified as N1 but re-staged as N2 had worse outcomes than the initial N1 patients, but similar to the initial N2 patients^6–8,22^. Thus, TD could serve as a supplement to N stage. However, the strategy had limitations as it ignored the exact number of TD and lymph nodes, especially when both pathological features were present^9^. Until now, the medical community remained divided on such integration. Research had demonstrated that at least 12 lymph nodes should be retrieved in a regional lymph node dissection specimen. Nonetheless, many patients had fewer than 12 lymph nodes removed, possibly due to factors such as low-position tumor, age, and others, which could potentially impact the prognosis. Increasing evidence suggested that in predicting prognosis, LNR was superior to the number of LNM^6,11–13^. In this study, through a retrospective analysis of stage III CRC cohorts, we demonstrated that TD-positive and H-LNR were associated with poorly differentiated tumors, T3-4 stage, presence of PNI, worse OS, and CSS.

Since 2004, fluorouracil, leucovorin and oxaliplatin (FOLFOX) and capecitabine and oxaliplatin (CAPOX) became the standard adjuvant therapy for stage III CRC^23,24^. A prospective study conducted by the IDEA concluded that a 3-month CAPOX regimen exhibited equivalent efficacy to a 6-month regimen, with reduced chemotherapy side effects^4^. Furthermore, the addition of TD to LNM may aid in determining the optimal adjuvant therapy duration^22^. Thus, the current TNM staging may be underestimated due to ignoring TD. Herein, we jointly analyzed TD and LNR, and found that the combined variable appeared to be more powerful than individual variable in predicting prognosis. Notably, when stratified by T/N risk, patients with TD-positive/H-LNR in the low-risk group had a worse prognosis than those with TD-negative/L-LNR in the high-risk group. As such, it is plausible that the combination variable of TD and LNR may serve as an effective supplement to the current TNM staging for stage III CRC.

CRC had distinct embryonic origins. The right colon derived from the midgut and the left colon and rectum derived from the hindgut. Numerous studies had described significant disparities in epidemiology, pathogenesis, genetic, and molecular pathways between right- and left-sided colon cancers^14,18,25^. However, the prognostic value of tumors on the left and right sides remained controversial. A study involving 77 978 subjects from the SEER database showed that right-sided tumors were associated with poorer prognoses^26^, which contradicted the results of another large-sample study^27^. Moreover, the anti-EGFR therapy appeared to be effective only for left-sided tumors^28^. We further analyzed the prognostic value of TD and LNR stratified by tumor laterality. In the training set, H-LNR exhibited a stronger association with right-sided colon cancer compared to left-sided tumors. Moreover, patients with TD-positive/H-LNR on the right side had the worst prognosis.

Interestingly, the prognostic value of TD and LNR was still confirmed in multivariable model adjusting for age, tumor size, laterality, grade, T stage, N stage, examined N, CEA, PNI, and chemotherapy. The combined variable of TD and LNR contributed the most to CSS. To provide clinicians with a useful tool for predicting patient outcomes, a nomogram was constructed based on significant factors from the multivariable analysis in the training set. The calibration curves demonstrated good accuracy in predicting 3-year and 5-year survival.

There are some limitations in our study. Firstly, retrospective design inevitably involves missing data, such as patients’ behaviors and overall health status, specific chemotherapy drugs, and details of follow-up care, which could potentially influence the prognosis. As an observational study, residual confounding cannot be excluded and thus limits our ability for causal inference. Future prospective studies are necessary to validate our findings. Secondly, the SEER database contains a certain proportion of cases with missing data that needed to be excluded, potentially weakening the representativeness of our study results. However, we obtained similar results in an independent external validation. Lastly, we did not consider the exact quantity of TD, but the number of TD has not been considered in the UICC 8th TNM staging either. Furthermore, the number of TD appeared to have no correlation with prognosis in the chemotherapy subgroup of patients^29^.

## Conclusions

Our study provides evidence that TD and LNR are independent prognostic factors in stage III CRC, particularly on right side. The combined variable of TD and LNR is a strong predictor of CSS, which could be used as a tool to assist in clinical decision-making.

## Ethical approval

The study was approved by the Ethics Committee of the Second Affiliated Hospital of Nanjing Medical University (IRB Approval No. 2020-092), and the Affiliated Yixing Hospital of Jiangsu University (IRB Approval No. 2022-158).

## Consent

Informed consent was waived by both committees due to the retrospective nature of the study.

## Sources of funding

This study was supported by the National Natural Science Foundation of China (81973127 and 81902925), the Outstanding Youth Fund of Jiangsu Natural Science Foundation (BK20230005), and the Key Discipline Construction Project of Jiangsu Provincial Health Commission (ZDXKA2016010).

## Author contribution

L.L.: data curation, formal analysis, software, and writing – original draft; J.J.: data curation, funding acquisition, methodology, and writing – original draft; X.X.G.: data curation, methodology, and writing – review and editing; Z.H.J. and J.C.L.: data curation, software, and writing – review and editing; J.W., J.T.Z., J.N.Y., F.Y.Z., and B.N.M.: data curation and writing – review and editing; Z.H.C. and J.Y.Z.: formal analysis, resources, and writing – review and editing; L.M. and G.Z.J.: funding acquisition, project administration, and writing – review & editing; D.H.: conceptualization, funding acquisition, supervision, and writing – review and editing.

## Conflicts of interest disclosure

The authors declare no conflicts of interest.

## Research registration unique identifying number (UIN)

1. Name of the registry: https://www.researchregistry.com/.
2. Unique identifying number or registration ID: researchregistry9354.
3. Hyperlink:https://www.researchregistry.com/browse-theregistry#home/registrationdetails/64c9b4aa5d2fa20028f6876e/.

## Guarantor

Lei Liu, MD.

## Data availability statement

The data used in this study were freely obtained from the SEER program (https://seer.cancer.gov/) and Chinese hospital databases. The original anonymous datasets are available from the corresponding authors on reasonable request.

## Provenance and peer review

Not commissioned, externally peer-reviewed.

## Supplementary Material
