# Supplementary material for: Prognostic value of tumor deposits and positive lymph node ratio in stage III colorectal cancer: a retrospective cohort study
Source: Int J Surg. 2024 Mar 18;110(6):3470–9. doi: 10.1097/JS9.0000000000001295 (PMC11175780; doi:10.1097/JS9.0000000000001295)
Supplement: Supplementary file 2 [file js9-110-3470-s002.docx]

**Prognostic value of tumor deposits and positive lymph node ratio in stage III colorectal cancer: a retrospective cohort study**

Lei Liu, MD^1,2†^, Jie Ji, MD^3†^, Xianxiu Ge, MD^1†^, Zuhong Ji, MSc^1^, Jiacong Li, MSc^4^, Jie Wu, MD^1^, Juntao Zhu, MSc^1^, Jianan Yao, MSc^1^, Fangyu Zhu, MSc^5^, Boneng Mao, MSc^2^, Zhihong Cao, MSc^2^, Jinyi Zhou, MD^5^, Lin Miao, MD^1*^, Guozhong Ji, MD^1*^, Dong Hang, PHD^4,6*^

**Supplementary Tables**

Supplementary Table 1. The prognostic significance of tumor deposits and lymph node ratio stratified by tumor laterality.

| Site of the tumor, variables | HR (95% CI)^a^ | *P*^a^ value | *P^b^* value for heterogeneity |
| --- | --- | --- | --- |
| **CSS** | | | |
| Training set |  |  |  |
| TD (positive versus negative) |  |  | 0.18 |
| Left | 1.80(1.68-1.93) | <0.001 |  |
| Right | 1.93(1.80-2.08) | <0.001 |  |
| LNR (high versus low) |  |  | <0.001 |
| Left | 2.47(2.28-2.68) | <0.001 |  |
| Right | 3.09(2.86-3.34) | <0.001 |  |
| Validation set |  |  |  |
| TD (positive versus negative) |  |  | 0.44 |
| Left | 1.74(1.23-2.46) | 0.002 |  |
| Right | 2.21(1.35-3.65) | 0.002 |  |
| LNR (high versus low) |  |  | 0.12 |
| Left | 3.13(2.20-4.45) | <0.001 |  |
| Right | 1.70(0.87-3.34) | 0.12 |  |
| **OS** | | | |
| Training set |  |  |  |
| TD (positive versus negative) |  |  | 0.52 |
| Left | 1.61(1.52-1.72) | <0.001 |  |
| Right | 1.66(1.56-1.77) | <0.001 |  |
| LNR (high versus low) |  |  | <0.001 |
| Left | 2.10(1.96-2.26) | <0.001 |  |
| Right | 2.55(2.38-2.73) | <0.001 |  |
| Validation set |  |  |  |
| TD (positive versus negative) |  |  | 0.63 |
| Left | 1.57(1.17-2.12) | 0.003 |  |
| Right | 1.79(1.14-2.82) | 0.012 |  |
| LNR (high versus low) |  |  | 0.25 |
| Left | 2.48(1.82-3.38) | <0.001 |  |
| Right | 1.68(0.93-3.02) | 0.084 |  |
| ^a^ Univariable Cox proportional hazards regression models. | | | |
| ^b^ *P* value for heterogeneity was assessed by the contrast test method. | | | |
| HR, hazard ratio; CI, confidence interval; TD, tumor deposit; LNR, lymph node ratio; CSS, cancer-specific survival; OS, overall survival. | | | |

Supplementary Table 2. Multivariable analyses of overall survival and cancer-specific survival in the validation set.

| Variables | OS | | CSS | |
| --- | --- | --- | --- | --- |
|  | HR (95% CI)^a^ | *P* value^a^ | HR (95% CI)^a^ | *P* value^a^ |
| Age | 1.03(1.02-1.05) | <0.001 | 1.03(1.02-1.05) | <0.001 |
| Laterality |  |  |  |  |
| Left | 1(Reference) |  | 1(Reference) |  |
| Right | 1.24(0.95-1.63) | 0.12 | 1.34(0.98-1.84) | 0.07 |
| Tumor size |  |  |  |  |
| ≤5cm | 1(Reference) |  | 1(Reference) |  |
| >5cm | 0.97(0.72-1.29) | 0.81 | 0.93(0.66-1.29) | 0.65 |
| Grade |  |  |  |  |
| Well/moderate | 1(Reference) |  | 1(Reference) |  |
| Poor/undifferentiated | 1.46(1.13-1.89) | 0.004 | 1.52(1.13-2.05) | 0.01 |
| T stage |  |  |  |  |
| T1-2 | 1(Reference) |  | 1(Reference) |  |
| T3-4 | 1.71(0.98-2.98) | 0.06 | 2.38(1.10-5.15) | 0.03 |
| N stage |  |  |  |  |
| N1(<4 nodes) | 1(Reference) |  | 1(Reference) |  |
| N2(≥4 nodes) | 1.29(0.94-1.77) | 0.12 | 1.32(0.90-1.92) | 0.15 |
| Examined N |  |  |  |  |
| <12 | 1(Reference) |  | 1(Reference) |  |
| ≥12 | 0.73(0.51-1.05) | 0.09 | 0.79(0.51-1.21) | 0.27 |
| CEA |  |  |  |  |
| Negative | 1(Reference) |  | 1(Reference) |  |
| Positive | 1.88(1.47-2.40) | <0.001 | 1.99(1.49-2.65) | <0.001 |
| PNI |  |  |  |  |
| Absent | 1(Reference) |  | 1(Reference) |  |
| Present | 1.21(0.88-1.66) | 0.23 | 1.05(0.72-1.51) | 0.82 |
| Chemotherapy |  |  |  |  |
| No | 1(Reference) |  | 1(Reference) |  |
| Yes | 0.94(0.73-1.22) | 0.66 | 0.95(0.71-1.29) | 0.75 |
| TD |  |  |  |  |
| Negative | 1(Reference) |  | 1(Reference) |  |
| Positive | 1.62(1.25-2.09) | <0.001 | 1.90(1.41-2.54) | <0.001 |
| LNR |  |  |  |  |
| Low | 1(Reference) |  | 1(Reference) |  |
| High | 1.60(1.08-2.36) | 0.02 | 2.01(1.29-3.15) | 0.002 |
| ^a^ Multivariable Cox proportional hazards regression model included age, laterality, tumor size, grade, T stage, N stage, examined N, CEA, PNI, chemotherapy, TD, and LNR. | | | | |
| CSS, cancer-specific survival; OS, overall survival; HR, hazard ratio; CI, confidence interval; Examined N, total examined lymph nodes; CEA, carcinoembryonic antigen; PNI, perineural invasion; TD, tumor deposit; LNR, lymph node ratio. | | | | |

Supplementary Table 3. Univariable and multivariable analyses of overall survival in the training set.

| Variables | Univariable^a^ | | Multivariable^b^ | |
| --- | --- | --- | --- | --- |
|  | HR (95% CI) | *P* value | HR (95% CI) | *P* value |
| Age | 1.04(1.04-1.04) | <0.001 | 1.03(1.03-1.03) | <0.001 |
| Gender |  |  |  |  |
| Female | 1(Reference) |  |  |  |
| Male | 1.04(1.00-1.08) | 0.05 |  |  |
| Laterality |  |  |  |  |
| Left | 1(Reference) |  | 1(Reference) |  |
| Right | 1.47(1.41-1.53) | <0.001 | 1.18(1.12-1.23) | <0.001 |
| Tumor number |  |  |  |  |
| 1 | 1(Reference) |  | 1(Reference) |  |
| >1 | 1.27(1.09-1.48) | 0.002 | 0.93(0.80-1.08) | 0.35 |
| Tumor size |  |  |  |  |
| ≤5cm | 1(Reference) |  | 1(Reference) |  |
| >5cm | 1.32(1.27-1.38) | <0.001 | 1.11(1.07-1.16) | <0.001 |
| Grade |  |  |  |  |
| Well/moderate | 1(Reference) |  | 1(Reference) |  |
| Poor/undifferentiated | 1.68(1.61-1.75) | <0.001 | 1.29(1.24-1.35) | <0.001 |
| T stage |  |  |  |  |
| T1-2 | 1(Reference) |  | 1(Reference) |  |
| T3-4 | 2.22(2.05-2.40) | <0.001 | 1.59(1.47-1.73) | <0.001 |
| N stage |  |  |  |  |
| N1(<4 nodes) | 1(Reference) |  | 1(Reference) |  |
| N2(≥4 nodes) | 1.67(1.60-1.74) | <0.001 | 1.39(1.32-1.46) | <0.001 |
| Examined N |  |  |  |  |
| <12 | 1(Reference) |  | 1(Reference) |  |
| ≥12 | 0.68(0.64-0.72) | <0.001 | 0.73(0.68-0.78) | <0.001 |
| CEA |  |  |  |  |
| Negative | 1(Reference) |  | 1(Reference) |  |
| Positive | 1.65(1.58-1.72) | <0.001 | 1.39(1.33-1.44) | <0.001 |
| PNI |  |  |  |  |
| Absent | 1(Reference) |  | 1(Reference) |  |
| Present | 1.55(1.48-1.63) | <0.001 | 1.36(1.30-1.43) | <0.001 |
| Neoadjuvant therapy |  |  |  |  |
| No | 1(Reference) |  | 1(Reference) |  |
| Yes | 0.91(0.85-0.98) | 0.01 | 1.18(1.09-1.27) | <0.001 |
| Chemotherapy |  |  |  |  |
| No | 1(Reference) |  | 1(Reference) |  |
| Yes | 0.36(0.35-0.38) | <0.001 | 0.47(0.45-0.50) | <0.001 |
| TD |  |  |  |  |
| Negative | 1(Reference) |  | 1(Reference) |  |
| Positive | 1.58(1.51-1.65) | <0.001 | 1.39(1.33-1.45) | <0.001 |
| LNR |  |  |  |  |
| Low | 1(Reference) |  | 1(Reference) |  |
| High | 2.29(2.18-2.40) | <0.001 | 1.63(1.53-1.74) | <0.001 |
| ^a^ Univariable Cox proportional hazards regression models. | | | | |
| ^b^ Multivariable Cox proportional hazards regression model included age, laterality, tumor number, tumor size, grade, T stage, N stage, examined N, CEA, PNI, neoadjuvant therapy, chemotherapy, TD, and LNR. | | | | |
| HR, hazard ratio; CI, confidence interval; Examined N, total examined lymph nodes; CEA, carcinoembryonic antigen; PNI, perineural invasion; TD, tumor deposit; LNR, lymph node ratio. | | | | |

Supplementary Table 4. Sensitivity analysis by additionally adjusting for histology, diagnosis year, and radiation in the training and validation sets.

|  | TD | | | LNR | | |
| --- | --- | --- | --- | --- | --- | --- |
|  | Negative | Positive | *P* value | Low | High | *P* value |
| **The training set** |  |  |  |  |  |  |
| CSS |  |  |  |  |  |  |
| No. of events (n=6685) | 4386 | 2299 |  | 5042 | 1643 |  |
| HR (95% CI)^a^ | 1(Reference) | 1.51(1.44-1.60) | <0.001 | 1(Reference) | 1.70(1.59-1.83) | <0.001 |
| OS |  |  |  |  |  |  |
| No. of events (n=9282) | 6386 | 2896 |  | 7302 | 1980 |  |
| HR (95% CI)^a^ | 1(Reference) | 1.41(1.34-1.47) | <0.001 | 1(Reference) | 1.60(1.51-1.71) | <0.001 |
| **The validation set** |  |  |  |  |  |  |
| CSS |  |  |  |  |  |  |
| No. of events (n=202) | 125 | 77 |  | 143 | 59 |  |
| HR (95% CI)^a^ | 1(Reference) | 1.56(1.09-2.24) | 0.02 | 1(Reference) | 2.14(1.28-3.56) | 0.004 |
| OS |  |  |  |  |  |  |
| No. of events (n=276) | 180 | 96 |  | 205 | 71 |  |
| HR (95% CI)^a^ | 1(Reference) | 1.41(1.04-1.92) | 0.03 | 1(Reference) | 1.67(1.07-2.61) | 0.02 |
| ^a^ Hazard ratios (HRs) and 95% confidence intervals (CIs) were estimated in the Cox proportional hazards regression model adjusting for age, laterality, tumor size, grade, T stage, N stage, examined N, CEA, PNI, chemotherapy, histology, diagnosis year, and radiation. TD, tumor deposit; LNR, lymph node ratio; CSS, cancer-specific survival; OS, overall survival. | | | | | | |

Supplementary Table 5. Univariable and multivariable analyses of cancer-specific survival in the training set without neoadjuvant therapy.

| Variables | Univariable^a^ | | Multivariable^b^ | |
| --- | --- | --- | --- | --- |
|  | HR (95% CI) | *P* value | HR (95% CI) | *P* value |
| Age | 1.03(1.03-1.03) | <0.001 | 1.02(1.02-1.02) | <0.001 |
| Gender |  |  |  |  |
| Female | 1(Reference) |  |  |  |
| Male | 1.00(0.95-1.05) | 0.98 |  |  |
| Laterality |  |  |  |  |
| Left | 1(Reference) |  | 1(Reference) |  |
| Right | 1.46(1.39-1.54) | <0.001 | 1.22(1.15-1.28) | <0.001 |
| Tumor number |  |  |  |  |
| 1 | 1(Reference) |  |  |  |
| >1 | 1.21(1.00-1.46) | 0.06 |  |  |
| Tumor size |  |  |  |  |
| ≤5cm | 1(Reference) |  | 1(Reference) |  |
| >5cm | 1.40(1.33-1.47) | <0.001 | 1.14(1.08-1.20) | <0.001 |
| Grade |  |  |  |  |
| Well/moderate | 1(Reference) |  | 1(Reference) |  |
| Poor/undifferentiated | 1.83(1.74-1.93) | <0.001 | 1.35(1.28-1.43) | <0.001 |
| T stage |  |  |  |  |
| T1-2 | 1(Reference) |  | 1(Reference) |  |
| T3-4 | 3.08(2.75-3.45) | <0.001 | 2.01(1.79-2.25) | <0.001 |
| N stage |  |  |  |  |
| N1(<4 nodes) | 1(Reference) |  | 1(Reference) |  |
| N2(≥4 nodes) | 2.08(1.98-2.19) | <0.001 | 1.57(1.48-1.67) | <0.001 |
| Examined N |  |  |  |  |
| <12 | 1(Reference) |  | 1(Reference) |  |
| ≥12 | 0.65(0.60-0.71) | <0.001 | 0.70(0.64-0.76) | <0.001 |
| CEA |  |  |  |  |
| Negative | 1(Reference) |  | 1(Reference) |  |
| Positive | 1.80(1.71-1.89) | <0.001 | 1.46(1.39-1.54) | <0.001 |
| PNI |  |  |  |  |
| Absent | 1(Reference) |  | 1(Reference) |  |
| Present | 1.77(1.67-1.87) | <0.001 | 1.42(1.34-1.51) | <0.001 |
| Chemotherapy |  |  |  |  |
| No | 1(Reference) |  | 1(Reference) |  |
| Yes | 0.41(0.39-0.43) | <0.001 | 0.49(0.46-0.52) | <0.001 |
| TD |  |  |  |  |
| Negative | 1(Reference) |  | 1(Reference) |  |
| Positive | 1.85(1.75-1.95) | <0.001 | 1.52(1.44-1.61) | <0.001 |
| LNR |  |  |  |  |
| Low | 1(Reference) |  | 1(Reference) |  |
| High | 2.80(2.64-2.96) | <0.001 | 1.74(1.62-1.87) | <0.001 |
| ^a^ Univariable Cox proportional hazards regression models. | | | | |
| ^b^ Multivariable Cox proportional hazards regression model included age, laterality, tumor size, grade, T stage, N stage, examined N, CEA, PNI, chemotherapy, TD and LNR. | | | | |
| HR, hazard ratio; CI, confidence interval; Examined N, total examined lymph nodes; CEA, carcinoembryonic antigen; PNI, perineural invasion; TD, tumor deposit; LNR, lymph node ratio. | | | | |

Supplementary Table 6. Univariable and multivariable analyses of overall survival in the training set without neoadjuvant therapy.

| Variables | Univariable^a^ | | Multivariable^b^ | |
| --- | --- | --- | --- | --- |
|  | HR (95% CI) | *P* value | HR (95% CI) | *P* value |
| Age | 1.04(1.04-1.05) | <0.001 | 1.03(1.03-1.03) | <0.001 |
| Gender |  |  |  |  |
| Female | 1(Reference) |  |  |  |
| Male | 1.03(0.99-1.08) | 0.15 |  |  |
| Laterality |  |  |  |  |
| Left | 1(Reference) |  | 1(Reference) |  |
| Right | 1.49(1.43-1.55) | <0.001 | 1.18(1.12-1.23) | <0.001 |
| Tumor number |  |  |  |  |
| 1 | 1(Reference) |  | 1(Reference) |  |
| >1 | 1.26(1.07-1.47) | 0.01 | 0.92(0.78-1.07) | 0.27 |
| Tumor size |  |  |  |  |
| ≤5cm | 1(Reference) |  | 1(Reference) |  |
| >5cm | 1.32(1.27-1.38) | <0.001 | 1.10(1.05-1.15) | <0.001 |
| Grade |  |  |  |  |
| Well/moderate | 1(Reference) |  | 1(Reference) |  |
| Poor/undifferentiated | 1.69(1.61-1.76) | <0.001 | 1.29(1.23-1.35) | <0.001 |
| T stage |  |  |  |  |
| T1-2 | 1(Reference) |  | 1(Reference) |  |
| T3-4 | 2.26(2.08-2.45) | <0.001 | 1.60(1.47-1.74) | <0.001 |
| N stage |  |  |  |  |
| N1(<4 nodes) | 1(Reference) |  | 1(Reference) |  |
| N2(≥4 nodes) | 1.67(1.6-1.74) | <0.001 | 1.39(1.32-1.47) | <0.001 |
| Examined N |  |  |  |  |
| <12 | 1(Reference) |  | 1(Reference) |  |
| ≥12 | 0.65(0.60-0.69) | <0.001 | 0.71(0.66-0.76) | <0.001 |
| CEA |  |  |  |  |
| Negative | 1(Reference) |  | 1(Reference) |  |
| Positive | 1.69(1.62-1.76) | <0.001 | 1.40(1.35-1.47) | <0.001 |
| PNI |  |  |  |  |
| Absent | 1(Reference) |  | 1(Reference) |  |
| Present | 1.53(1.46-1.61) | <0.001 | 1.34(1.27-1.41) | <0.001 |
| Chemotherapy |  |  |  |  |
| No | 1(Reference) |  | 1(Reference) |  |
| Yes | 0.35(0.33-0.36) | <0.001 | 0.46(0.44-0.48) | <0.001 |
| TD |  |  |  |  |
| Negative | 1(Reference) |  | 1(Reference) |  |
| Positive | 1.61(1.53-1.68) | <0.001 | 1.41(1.34-1.47) | <0.001 |
| LNR |  |  |  |  |
| Low | 1(Reference) |  | 1(Reference) |  |
| High | 2.30(2.19-2.43) | <0.001 | 1.63(1.53-1.74) | <0.001 |
| ^a^ Univariable Cox proportional hazards regression models. | | | | |
| ^b^ Multivariable Cox proportional hazards regression model included age, laterality, tumor number, tumor size, grade, T stage, N stage, examined N, CEA, PNI, chemotherapy, TD, and LNR. | | | | |
| HR, hazard ratio; CI, confidence interval; Examined N, total examined lymph nodes; CEA, carcinoembryonic antigen; PNI, perineural invasion; TD, tumor deposit; LNR, lymph node ratio. | | | | |

Supplementary Table 7**.** Association of TD and LNR with mortality according to the year of CRC diagnosis in the training and validation sets.

|  | TD | | *P* value | *P* for  heterogeneity | LNR | | *P* value | *P* for  heterogeneity |
| --- | --- | --- | --- | --- | --- | --- | --- | --- |
|  | Negative | Positive |  |  | Low | High |  |  |
| **The training set** | | | | | | | | |
| CSS | | | | | | | | |
| 2010-2012, HR (95% CI)^a^ | 1(Reference) | 1.56(1.43-1.69) | <0.001 | 0.58 | 1(Reference) | 1.55(1.39-1.72) | <0.001 | 0.03 |
| 2013-2016, HR (95% CI)^a^ | 1(Reference) | 1.47(1.36-1.59) | <0.001 |  | 1(Reference) | 1.89(1.70-2.09) | <0.001 |  |
| After 2016, HR (95% CI)^a^ | 1(Reference) | 1.48(1.26-1.73) | <0.001 |  | 1(Reference) | 1.73(1.38-2.16) | <0.001 |  |
| OS | | | | | | | | |
| 2010-2012, HR (95% CI)^a^ | 1(Reference) | 1.44(1.34-1.55) | <0.001 | 0.49 | 1(Reference) | 1.49(1.36-1.64) | <0.001 | 0.06 |
| 2013-2016, HR (95% CI)^a^ | 1(Reference) | 1.38(1.29-1.47) | <0.001 |  | 1(Reference) | 1.75(1.59-1.91) | <0.001 |  |
| After 2016, HR (95% CI)^a^ | 1(Reference) | 1.33(1.16-1.53) | <0.001 |  | 1(Reference) | 1.62(1.32-1.98) | <0.001 |  |
| **The validation set** | | | | | | | | |
| CSS | | | | | | | | |
| 2013-2016, HR (95% CI)^a^ | 1(Reference) | 2.10(1.39-3.15) | <0.001 | 0.54 | 1(Reference) | 2.15(1.06-4.33) | 0.03 | 0.99 |
| After 2016, HR (95% CI)^a^ | 1(Reference) | 1.74(1.12-2.69) | 0.01 |  | 1(Reference) | 2.16(1.20-3.89) | 0.01 |  |
| OS | | | | | | | | |
| 2013-2016, HR (95% CI)^a^ | 1(Reference) | 1.77(1.24-2.51) | 0.001 | 0.53 | 1(Reference) | 1.66(0.91-3.04) | 0.10 | 0.90 |
| After 2016, HR (95% CI)^a^ | 1(Reference) | 1.49(1.01-2.20) | 0.04 |  | 1(Reference) | 1.75(1.04-2.95) | 0.03 |  |
| ^a^ Hazard ratios (HRs) and 95% confidence intervals (CIs) were estimated in the Cox proportional hazards regression model adjusting for age, laterality, tumor size, grade, T stage, N stage, examined N, CEA, PNI, and chemotherapy. TD, tumor deposit; LNR, lymph node ratio; CSS, cancer-specific survival; OS, overall survival. | | | | | | | | |

**Supplementary figures**

**
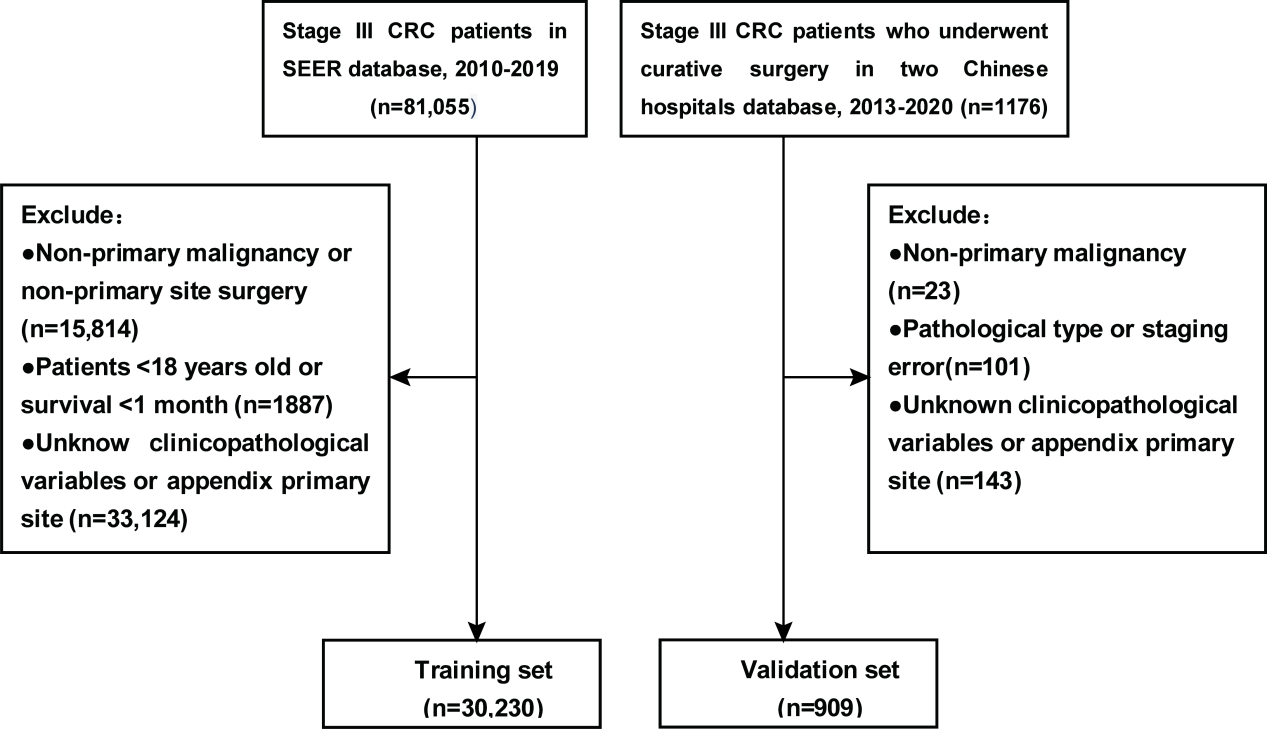
**

**Figure S1** Flow chart of the study.


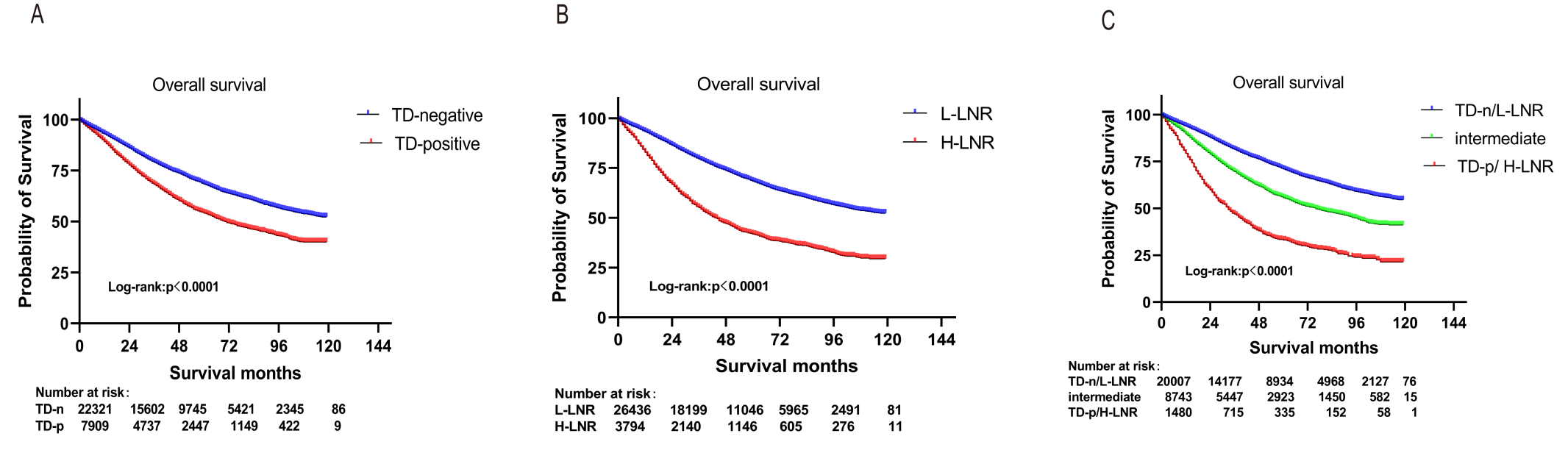


**Figure S2** Kaplan-Meier plots of OS by TD (A), LNR (B) and the combined variable of TD and LNR (C) in the training set. OS, overall survival; TD, tumor deposit; TD-n, TD-negative; TD-p, TD-positive; LNR, lymph node ratio; L-LNR, low LNR; H-LNR, high LNR.

**
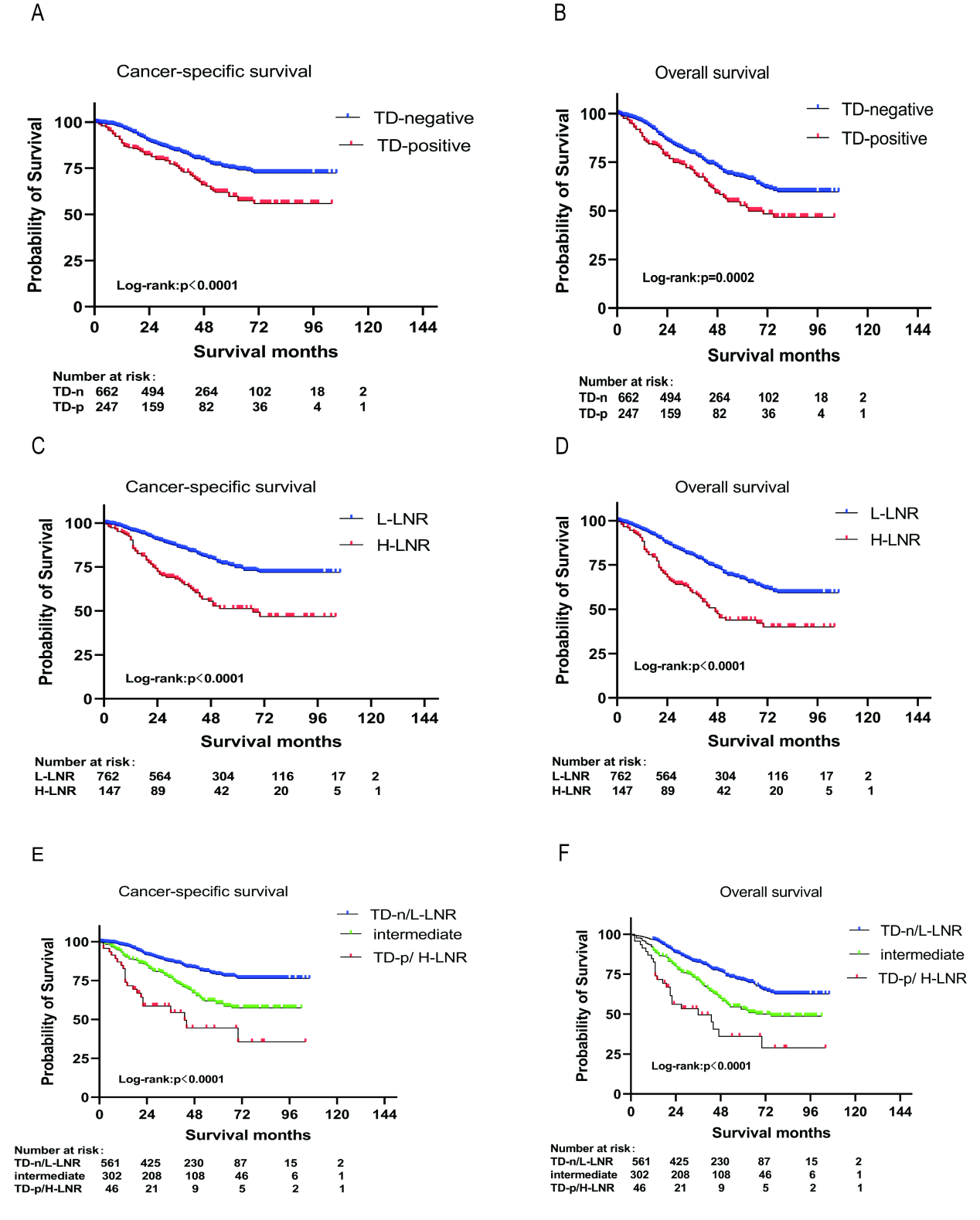
Figure S3** Kaplan-Meier plots of CSS and OS by TD (A and B, respectively), LNR (C and D, respectively), and their combined variable (E and F, respectively) in the validation set. CSS, cancer-specific survival; OS, overall survival; TD, tumor deposit; TD-n, TD-negative; TD-p, TD-positive; LNR, lymph node ratio; L-LNR, low LNR; H-LNR, high LNR.


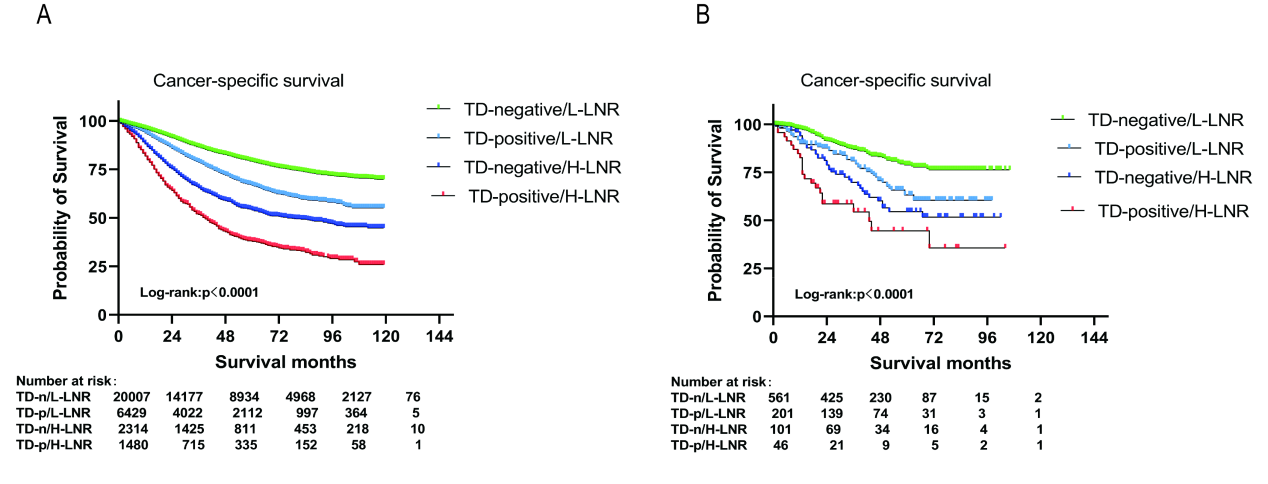


**Figure S4** Kaplan-Meier plots of CSS by further categorizing the intermediate group into TD-positive/L-LNR and TD-negative/H-LNR subgroups in the training (A) and validation (B) sets. CSS, cancer-specific survival; TD, tumor deposit; TD-n, TD-negative; TD-p, TD-positive; LNR, lymph node ratio; L-LNR, low LNR; H-LNR, high LNR.


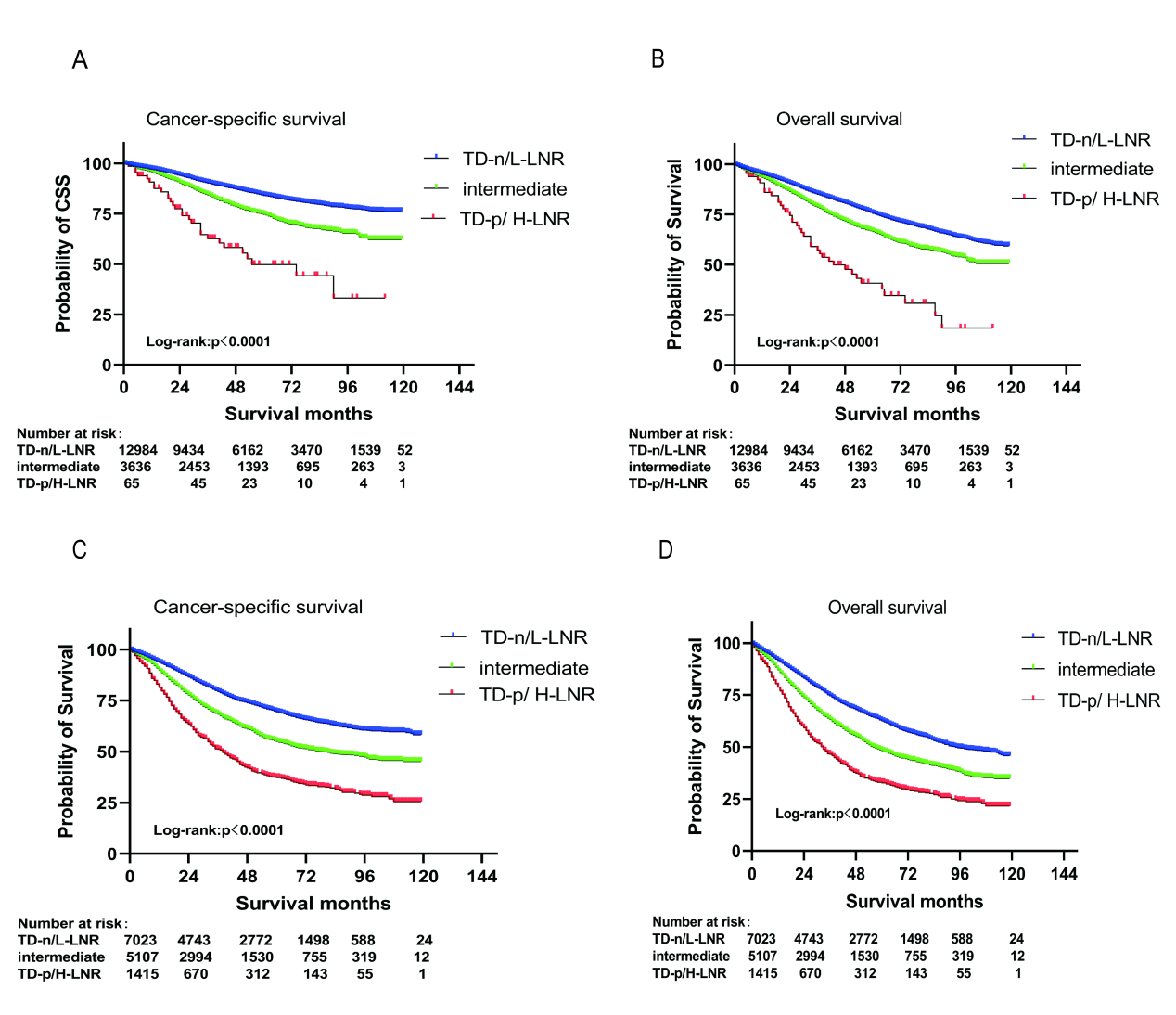


**Figure S5** Kaplan-Meier plots of CSS and OS by the combined variable of TD and LNR in low-risk (T1-3 N1) (A and B, respectively) and high-risk (T4 and/or N2) (C and D, respectively) groups in the training set. CSS, cancer-specific survival; OS, overall survival; TD, tumor deposit; TD-n, TD-negative; TD-p, TD-positive; LNR, lymph node ratio; L-LNR, low LNR; H-LNR, high LNR.


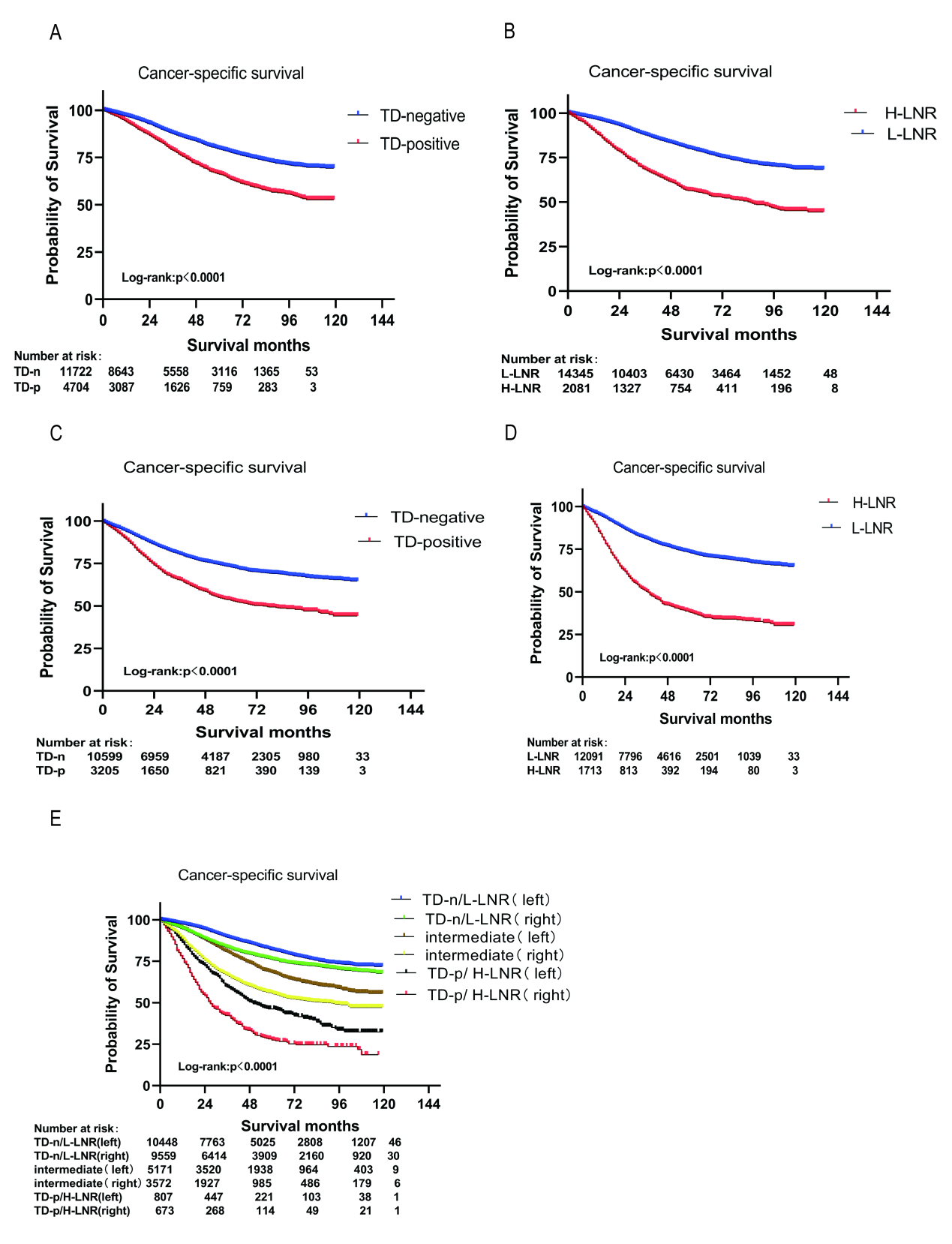


**Figure S6** Kaplan-Meier plots of CSS by TD and LNR in left-sided (A and B, respectively) and right-sided subgroups (C and D, respectively), and the combined variable of TD and LNR based on tumor laterality (E) in the training set. CSS, cancer-specific survival; TD, tumor deposit; TD-n, TD-negative; TD-p, TD-positive; LNR, lymph node ratio; L-LNR, low LNR; H-LNR, high LNR.
